# Idiopathic Intracranial Hypertension in a Non-obese Male Patient: A Case Report

**DOI:** 10.7759/cureus.102170

**Published:** 2026-01-23

**Authors:** Anas E Ahmed, Abdulmajeed F Alharbi, Muhannad M Alharbi, Ayah F Albalawai, Anas Y Shaheen

**Affiliations:** 1 Department of Community Medicine, Jazan University, Jazan, SAU; 2 College of Medicine, Tanta University, Tanta, EGY; 3 College of Medicine and Surgery, Umm Al-Qura University, Makkah, SAU; 4 College of Medicine, Tabuk University, Tabuk, SAU; 5 College of Medicine and Health Sciences, Abdulatif Alhamad University of Technology, Merowe, SDN

**Keywords:** acetazolamide, atypical presentation, elevated intracranial pressure, headache, idiopathic intracranial hypertension, lumbar puncture, neuroimaging, papilledema, visual disturbances

## Abstract

Idiopathic intracranial hypertension (IIH) is a disorder characterized by elevated intracranial pressure without an identifiable structural or secondary cause and is classically seen in obese women of childbearing age. We report a case of a 34-year-old non-obese male patient who presented with a six-month history of progressively worsening daily headaches, transient visual obscurations, and intermittent pulsatile tinnitus. Neurological examination revealed bilateral papilledema with preserved visual acuity, while laboratory investigations were unremarkable. Neuroimaging with computed tomography (CT) and magnetic resonance imaging (MRI) demonstrated features suggestive of raised intracranial pressure, including partial empty sella, posterior globe flattening, perioptic subarachnoid space distension, and optic nerve tortuosity, without evidence of mass lesion or venous sinus thrombosis. Lumbar puncture confirmed an elevated opening pressure of 32 cm H₂O with normal cerebrospinal fluid composition. A diagnosis of IIH was established after exclusion of secondary causes. The patient was managed conservatively with acetazolamide and symptomatic therapy, resulting in significant improvement in headache frequency and resolution of visual disturbances over three months, with stabilization of visual fields. This case highlights that IIH can occur in atypical populations, including non-obese men, and underscores the importance of early recognition, comprehensive evaluation, and timely intervention to prevent visual morbidity.

## Introduction

Idiopathic intracranial hypertension (IIH) is a disorder characterized by elevated intracranial pressure in the absence of an identifiable intracranial mass lesion, hydrocephalus, infection, or vascular abnormality, with normal cerebrospinal fluid (CSF) composition [1]. The condition predominantly affects obese women of childbearing age, with an estimated incidence of 1-3 per 100,000 in the general population and up to 20 per 100,000 among obese women [1,2]. The pathophysiology of IIH remains incompletely understood, but proposed mechanisms include impaired CSF absorption, increased cerebral venous pressure, and dysregulation of intracranial fluid dynamics [1-3]. Clinically, IIH commonly presents with headaches, visual disturbances, pulsatile tinnitus, and papilledema and carries a significant risk of permanent visual impairment if not recognized and treated promptly [2,3].

Although IIH is classically associated with obesity and female sex, it can occur in atypical populations, including non-obese men, where diagnosis may be delayed due to lower clinical suspicion [1-5]. Emerging evidence suggests that IIH in men may follow a more aggressive course with a higher risk of visual loss, underscoring the importance of early identification and careful monitoring [4,5]. Reporting such atypical cases is essential to broaden clinical awareness, highlight diagnostic challenges, and reinforce the need for thorough evaluation of raised intracranial pressure regardless of demographic risk factors. This case contributes to the limited literature on IIH in non-obese male patients and emphasizes adherence to standardized diagnostic and reporting guidelines.

## Case presentation

A 34-year-old male patient with no significant past medical history presented to the neurology outpatient clinic with a six-month history of progressively worsening headaches and intermittent visual disturbances. The headaches were daily, diffuse, pressure-like, more prominent in the retro-orbital and frontal regions, and exacerbated by Valsalva maneuvers, bending forward, and early morning hours. Pain intensity was rated as 7/10 and partially relieved by over-the-counter analgesics. The patient also reported transient visual obscurations lasting a few seconds, which occurred several times per day, particularly when standing, along with intermittent pulsatile tinnitus.

There was no history of diplopia, persistent visual loss, nausea, vomiting, seizures, focal neurological deficits, fever, head trauma, or altered level of consciousness. He denied recent weight gain, use of vitamin A derivatives, tetracyclines, corticosteroids, or hormonal therapy. There was no history suggestive of endocrine disorders, chronic kidney disease, anemia, sleep apnea, or systemic inflammatory disease. Family history was non-contributory, and he was a lifelong non-smoker with no alcohol or illicit drug use. His body mass index was 23.1 kg/m² (normal weight).

On physical examination, the patient was alert, oriented, and in no acute distress. Vital signs were within normal limits, including blood pressure of 122/78 mmHg. General examination revealed no signs of obesity, cushingoid features, or systemic illness. Neurological examination showed intact higher mental functions and cranial nerves, except for bilateral papilledema noted on fundoscopic examination, more pronounced on the right side, with blurred optic disc margins and hyperemia. Visual acuity was 6/6 in both eyes, and color vision was preserved. Visual field testing by confrontation suggested mild peripheral constriction, later confirmed by automated perimetry. Extraocular movements were full, with no evidence of sixth nerve palsy. Motor, sensory, cerebellar, and gait examinations were unremarkable, and deep tendon reflexes were normal. There were no meningeal signs.

Initial laboratory investigations, including complete blood count, renal and liver function tests, serum electrolytes, thyroid function tests, inflammatory markers, and coagulation profile, were within normal ranges. Hemoglobin level was 14.6 g/dL, excluding anemia as a contributing factor. Serologic tests for autoimmune and infectious etiologies, including antinuclear antibodies and human immunodeficiency virus (HIV), were negative (Table 1).

**Table 1:** Laboratory investigations of the patient at presentation Laboratory parameters including hematologic, renal, liver, electrolyte, thyroid, inflammatory, coagulation, and cerebrospinal fluid (CSF) studies are shown with their respective results, units, and reference ranges. CSF analysis was performed during diagnostic lumbar puncture in the lateral decubitus position. ESR: erythrocyte sedimentation rate; CRP: C-reactive protein; PT: prothrombin time; INR: international normalized ratio; aPTT: activated partial thromboplastin time; TSH: thyroid-stimulating hormone; ALT: alanine aminotransferase; AST: aspartate aminotransferase; SGPT: serum glutamate-pyruvate transaminase; SGOT: serum glutamate-oxaloacetate transaminase; cm H₂O: centimeters of water

Parameter	Result	Reference range
Hemoglobin	14.6	13.5–17.5 g/dL
Hematocrit	44	41%–53%
White blood cell count	6.2	4.0–11.0 × 10³/µL
Neutrophils	58	40%–70%
Lymphocytes	32	20%–45%
Monocytes	6	2%–10%
Eosinophils	3	1%–6%
Platelet count	230	150–450 × 10³/µL
Blood urea nitrogen	14	7–20 mg/dL
Creatinine	0.9	0.7–1.3 mg/dL
AST (SGOT)	22	10–40 U/L
ALT (SGPT)	25	7–56 U/L
Alkaline phosphatase	78	45–115 U/L
Total bilirubin	0.9	0.2–1.2 mg/dL
Direct bilirubin	0.2	0.0–0.3 mg/dL
Albumin	4.3	3.5–5.0 g/dL
Sodium	139	135–145 mmol/L
Potassium	4.2	3.5–5.0 mmol/L
Chloride	103	98–107 mmol/L
Bicarbonate	24	22–28 mmol/L
TSH	2.1	0.4–4.0 µIU/mL
Free T4	1.2	0.8–1.8 ng/dL
Free T3	3.1	2.3–4.2 pg/mL
ESR	10	0–20 mm/hr
CRP	3	0–5 mg/L
PT	12	11–14 sec
INR	1	0.8–1.2
aPTT	32	25–35 sec

**Figure 1:**
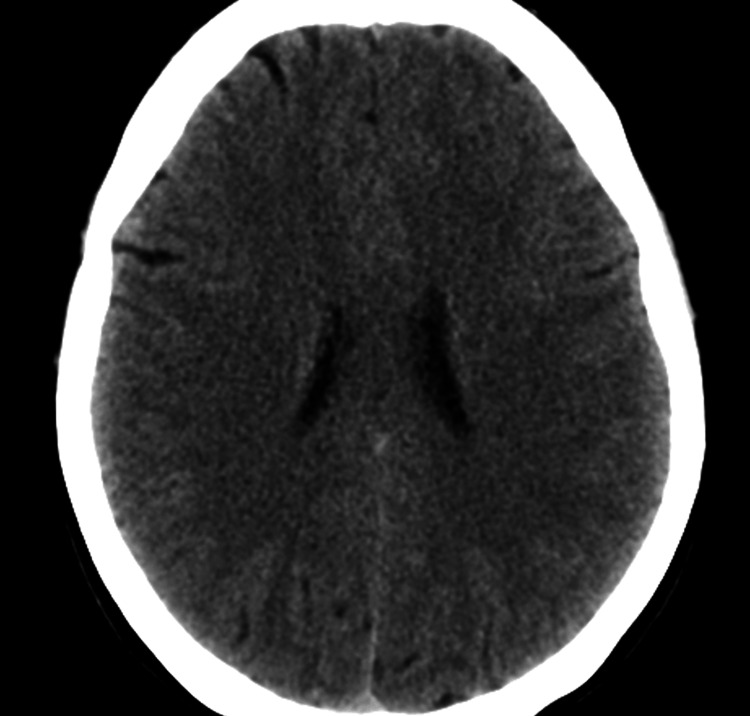
Normal non-contrast CT of the brain Axial non-contrast computed tomography (CT) image of the brain demonstrates no acute intracranial abnormalities. There is no evidence of intracranial hemorrhage, mass lesion, hydrocephalus, or midline shift. The ventricles and sulci are normal in size and configuration.

**Figure 2:**
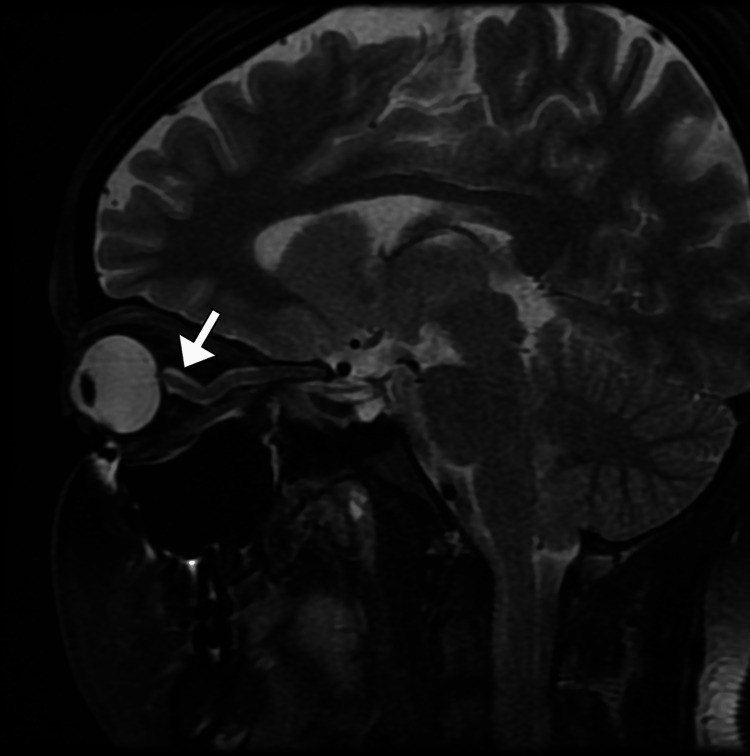
Dilated perioptic subarachnoid space on T2-weighted MRI Sagittal T2-weighted magnetic resonance image (MRI) of the orbit shows dilatation of the perineural (perioptic) subarachnoid space surrounding the optic nerve (arrow). This finding reflects increased cerebrospinal fluid pressure along the optic nerve sheath and is a characteristic imaging feature associated with idiopathic intracranial hypertension.

**Figure 3:**
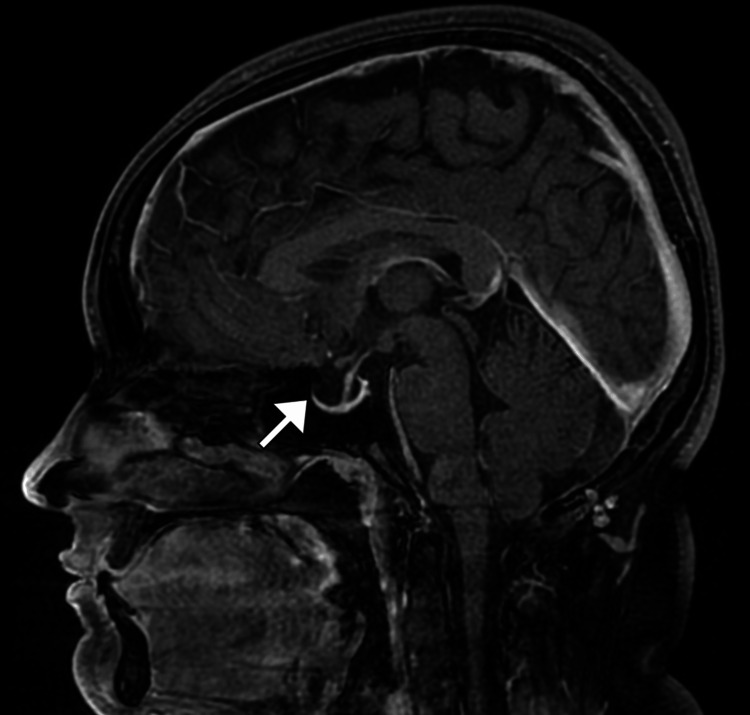
Nearly empty sella on contrast-enhanced T1-weighted MRI Sagittal contrast-enhanced T1-weighted magnetic resonance image (MRI) demonstrates an almost empty sella turcica (arrow), with flattening of the pituitary gland against the sellar floor. This appearance is a recognized radiologic sign of chronically elevated intracranial pressure and supports the diagnosis of idiopathic intracranial hypertension in the appropriate clinical context.

A non-contrast computed tomography (CT) scan of the brain demonstrated no intracranial mass lesion, hemorrhage, hydrocephalus, or structural abnormality (Figure 1). Subsequently, magnetic resonance imaging (MRI) of the brain with and without contrast revealed features suggestive of raised intracranial pressure, including posterior flattening of the globes, mild distension of the perioptic subarachnoid spaces (Figure 2), vertical tortuosity of the optic nerves, and partial empty sella (Figure 3). There was no evidence of venous sinus thrombosis, space-occupying lesion, or meningeal enhancement. Magnetic resonance venography (MRV) showed patent dural venous sinuses without stenosis or thrombosis.

Given the radiological findings and clinical presentation, a diagnostic lumbar puncture was performed in the lateral decubitus position under sterile conditions. Opening CSF pressure was elevated at 32 cm H₂O. CSF analysis revealed clear, colorless fluid with normal cell count, glucose, and protein levels, and no organisms on Gram stain or culture. Following the removal of 20 mL of CSF, the patient reported a transient improvement in headache severity.

Based on the presence of symptoms and signs of raised intracranial pressure, elevated opening pressure with normal CSF composition, normal neuroimaging excluding secondary causes, and absence of alternative etiologies, a diagnosis of IIH was established. The patient was initiated on medical management with oral acetazolamide at a dose of 500 mg twice daily, titrated upward as tolerated, along with dietary counseling to maintain a healthy weight and reduce sodium intake. Analgesics were prescribed for symptomatic relief of headaches. Ophthalmology consultation was obtained, and baseline automated visual field testing and optical coherence tomography (OCT) were performed for monitoring. During the hospital course, the patient demonstrated gradual improvement in headache frequency and severity over one week, with no progression of visual symptoms. He was discharged in stable condition with instructions for close outpatient follow-up.

At the three-month follow-up, the patient reported a significant reduction in headache intensity and frequency, with complete resolution of pulsatile tinnitus and transient visual obscurations. Follow-up visual field testing demonstrated stabilization without further deficits. Acetazolamide was continued at a maintenance dose, and ongoing monitoring by neurology and ophthalmology was planned.

## Discussion

IIH represents a diagnostic and therapeutic challenge, particularly when it occurs in populations outside the classic demographic profile. The present case highlights IIH in a non-obese male patient, an atypical presentation that underscores the importance of maintaining a high index of suspicion for this condition regardless of sex or body habitus. While obesity and female sex are well-established risk factors [5-8], accumulating evidence indicates that IIH can occur across a broader clinical spectrum, and atypical cases may be underrecognized or diagnosed later in the disease course, potentially increasing the risk of irreversible visual morbidity.

The pathophysiology of IIH remains incompletely elucidated [1-4]. Proposed mechanisms include impaired CSF absorption at the arachnoid granulations, increased cerebral venous sinus pressure, abnormalities in sodium and water regulation, and altered intracranial compliance [2,6]. In obese patients, increased intra-abdominal and intrathoracic pressures and hormonal factors such as adipokines have been implicated [7,8]. However, in non-obese men, alternative or overlapping mechanisms may predominate, including subtle venous outflow abnormalities, genetic susceptibility, or dysregulation of CSF dynamics independent of adiposity [2-5]. The absence of venous sinus thrombosis or significant stenosis on MRV in this case supports the diagnosis of truly idiopathic disease and highlights that raised intracranial pressure may occur even in the absence of identifiable structural or vascular abnormalities [1,4].

Clinically, headache remains the most common presenting symptom of IIH, often with features suggestive of raised intracranial pressure, as seen in this patient [3,4]. Transient visual obscurations, pulsatile tinnitus, and papilledema are key diagnostic clues and should prompt urgent neuro-ophthalmologic evaluation [6,7]. Notably, several studies have suggested that men with IIH may present with fewer headache symptoms but more severe or rapidly progressive visual impairment compared with women [2-6]. Although our patient had preserved visual acuity at presentation, the presence of bilateral papilledema and visual field constriction underscores the need for early diagnosis and close ophthalmologic surveillance in this population.

Neuroimaging plays a critical role in excluding secondary causes of intracranial hypertension and in supporting the diagnosis of IIH [1-8]. Characteristic MRI findings, including partial empty sella, posterior globe flattening, perioptic subarachnoid space distension, and optic nerve tortuosity, were present in this case and are increasingly recognized as supportive radiological markers [3,5]. While these findings are not pathognomonic, their presence in the appropriate clinical context strengthens diagnostic confidence [4,5]. Measurement of elevated CSF opening pressure with normal CSF composition remains essential and continues to be a cornerstone of diagnosis, in accordance with established diagnostic criteria [4-7].

Management of IIH aims to alleviate symptoms, preserve visual function, and reduce intracranial pressure [1-7]. Acetazolamide remains the first-line medical therapy, supported by evidence demonstrating its efficacy in reducing papilledema and improving visual outcomes [3,6]. The favorable clinical and ophthalmologic response observed in this patient reinforces the role of early medical therapy even in atypical cases [5,7]. Although weight loss is a key therapeutic target in obese patients, its role in non-obese individuals is less clear, and management should focus on pharmacologic therapy and vigilant monitoring [3,6,8]. Surgical interventions, such as optic nerve sheath fenestration or CSF diversion procedures, are reserved for refractory cases or those with rapidly progressive visual loss and were not required in this patient due to clinical improvement with conservative measures [5,8].

## Conclusions

This case highlights that IIH can occur in non-obese male patients and should be considered in any individual presenting with symptoms and signs of raised intracranial pressure and papilledema, irrespective of traditional risk factors. The key take-home message is that reliance on classic demographic profiles may delay diagnosis and increase the risk of preventable visual impairment. Comprehensive clinical evaluation, appropriate neuroimaging, and confirmation with elevated CSF opening pressure are essential for timely diagnosis, while early initiation of medical therapy and close ophthalmologic follow-up can lead to favorable outcomes. Reporting atypical presentations of IIH is crucial to enhancing clinical awareness, reducing diagnostic bias, and improving patient care.
